# Role of a Novel I1781T Mutation and Other Mechanisms in Conferring Resistance to Acetyl-CoA Carboxylase Inhibiting Herbicides in a Black-Grass Population

**DOI:** 10.1371/journal.pone.0069568

**Published:** 2013-07-25

**Authors:** Shiv Shankhar Kaundun, Sarah-Jane Hutchings, Richard P. Dale, Eddie McIndoe

**Affiliations:** Syngenta, Jealott’s Hill International Research Centre, Biological Sciences, Bracknell, United Kingom; Nanjing Agricultural University, China

## Abstract

**Background:**

Knowledge of the mechanisms of herbicide resistance is important for designing long term sustainable weed management strategies. Here, we have used an integrated biology and molecular approach to investigate the mechanisms of resistance to acetyl-CoA carboxylase inhibiting herbicides in a UK black-grass population (BG2).

**Methodology/Principal Findings:**

Comparison between BG2 phenotypes using single discriminant rates of herbicides and genotypes based on ACCase gene sequencing showed that the I1781L, a novel I1781T, but not the W2027C mutations, were associated with resistance to cycloxydim. All plants were killed with clethodim and a few individuals containing the I1781L mutation were partially resistant to tepraloxydim. Whole plant dose response assays demonstrated that a single copy of the mutant T1781 allele conferred fourfold resistance levels to cycloxydim and clodinafop-propargyl. In contrast, the impact of the I1781T mutation was low (Rf = 1.6) and non-significant on pinoxaden. BG2 was also characterised by high levels of resistance, very likely non-target site based, to the two cereal selective herbicides clodinafop-propargyl and pinoxaden and not to the poorly metabolisable cyclohexanedione herbicides. Analysis of 480 plants from 40 cycloxydim resistant black grass populations from the UK using two very effective and high throughput dCAPS assays established for detecting any amino acid changes at the 1781 ACCase codon and for positively identifying the threonine residue, showed that the occurrence of the T1781 is extremely rare compared to the L1781 allele.

**Conclusion/Significance:**

This study revealed a novel mutation at ACCase codon position 1781 and adequately assessed target site and non-target site mechanisms in conferring resistance to several ACCase herbicides in a black-grass population. It highlights that over time the level of suspected non-target site resistance to some cereal selective ACCase herbicides have in some instances surpassed that of target site resistance, including the one endowed by the most commonly encountered I1781L mutation.

## Introduction

Black-grass (*Alopecurus myosuroides* Huds.) is one of the most problematic weeds in North Western Europe [1]. It is a particularly challenging pest in autumn sown crops as most seeds germinate during this period [2]. Once established it is difficult to eliminate as it sheds seeds before the harvest, thus constantly replenishing the seed bank [3]. Though relatively less competitive than species such as wild oats, it can occur at high densities causing important losses in crop yield. On average 12 black grass plants per m^2^ can cause a yield reduction of 5% in winter cereals in the UK [4].

The most preferred method of black-grass control in arable crops is via the use of herbicides such as inhibitors of acetolactate synthase and acetyl-CoA carboxylase (ACCase). Of interest here, the ACCase herbicides are classified into aryloxyphenoxypropionates (FOPs), cyclohexanediones (DIMs) and phenylpyrazolin (DEN) based on their chemical structures [5], [6]. In all cases they bind to the carboxyltransferase domain of chloroplastic ACCase of most grass weeds with little to no action on the cytoplasmic isoform [7]. In so doing they inhibit the formation of malonyl-CoA, depleting the cells of important fatty acids, leading to rapid necrosis and plant death [8], [9]. Their selective action on grass weeds is attributable to the dissimilar structures of plastidic ACCase in the different plant groups. In Poaceae, acetyl-CoA carboxylase is homomeric with the biotin carboxyl carrier protein (BCCP), biotin carboxylase (BC) and carboxyl transferase α and β subdomains located on a single polypeptide. In contrast, the subunits are encoded by four different genes that are co-ordinately expressed to form a functional enzyme in other plant species [10].

Over time, the extensive use of ACCase herbicides has resulted in the evolution of resistance in the major grass weeds worldwide [11]. The first case of resistance to ACCase herbicides in black-grass was reported as early as 1982 in the UK. To date, resistance is widespread and affects most arable farms infested with black-grass [12]. Black-grass resistance to ACCase herbicides is also relatively common in France and is a growing problem in Germany, Belgium, Turkey, the Netherlands and Italy [13], [14].

Two different mechanisms account for resistance to ACCase herbicides. Resistance can be due to subtle amino acid changes in the ACCase, resulting in reduced sensitivities to the toxophores. In black-grass the I1781L, W2027C, I2041N, D2078G and G2096A mutations have been reported to confer varying levels of resistance to ACCase inhibiting herbicides [15], [16]. Recently, a comprehensive survey across its geographical range has shown that non-target site resistance to ACCase herbicides is more widespread than target site mechanisms [13]. The genetic basis of non-target site resistance appears to be very complex and governed by up to seven different loci [17]. Two sets of proteins involved in xenobiotic metabolism are associated with non-target site resistance to ACCase herbicides, namely cytochrome p450 and gluthathione *S*-transferase (G*S*T) enzymes [18]. In addition to their herbicide degrading properties, G*S*T enzymes from some multiple herbicide resistant black-grass populations have been shown to exhibit peroxidase activities, protecting the plants from the noxious effects of reactive oxygen radicals following herbicide treatment [19]. The complexity associated with G*S*T based metabolism is further exacerbated as the expression of the detoxifying enzymes varies with plant growth stages and environmental conditions [20].

In the UK, whilst black-grass resistance to extensively used cereal selective ACCase herbicides is widespread, the poorly metabolisable DIM herbicides which are often employed in rotational dicotyledonous crops are less affected and estimated to occur in around half of the populations resistant to FOP herbicides [21]. Here we have investigated the molecular basis of cycloxydim resistance in a black-grass population from the UK and determined its cross resistance to a number of commonly used ACCase herbicides, paying particular attention to estimating the level of resistance conferred by the different target site mutations and non-target site resistance mechanisms contained in this population. Additionally, we have developed two robust DNA markers following the identification of a novel mutation at ACCase codon position 1781.

## Materials and Methods

### Materials

Seeds from the suspected resistant black-grass population BG2 were sampled from a wheat field located in Buckinghamshire, UK. Over the years the field was treated with a range of ACCase herbicides applied in small grain cereal and rotational dicotyledonous crops. A standard sensitive population (STD1), used for comparison in all biological and molecular studies, was sourced from Herbiseed (Twyford, UK).

Additionally, 40 cycloxydim resistant black-grass populations collected from across the UK between 2002 and 2009 were analysed to estimate the relative frequency of a novel mutation identified at ACCase codon position 1781.

"No specific permissions were required for the location where the ryegrass seeds were collected. This study did not involve any endangered or protected species”.

### Methods

#### Confirmation of resistance to a DIM, FOP and DEN herbicides

Around 50 seeds each of BG2 and STD1 were sown and grown at the 2-leaf stage in 1 litre pots containing peat and compost in 1∶1 ratio. The pots were maintained and fertilised as necessary in controlled glasshouse conditions at 24°C/16 hr day, 18°C night, 65% relative humidity, and a photon flux density of approximately 250 µmol quanta m^–2^ s^–1^. Three replicate pots each of suspected resistant (BG2) and sensitive (STD1) populations were sprayed with the recommended field rates of cycloxydim (200 g ai/ha), clodinafop-propargyl (60 g ai/ha) and pinoxaden (60 g ai/ha) (a.i.: active ingredient).

#### Selection of cycloxydim sensitive and resistant plants and ACCase gene sequencing

The BG2 and STD1 plants were sown and grown as described above. At the one-leaf stage 48 BG2 and 16 STD1 plants were individually transplanted in single pots and grown to the two-leaf stage. The plants were sprayed with the recommended field rate of cycloxydim and survival was recorded 21 days after treatment. Prior to herbicide application, a 1-cm leaf tip segment was harvested from each of the 48 BG2 and 16 STD1 plants for sequencing a DNA fragment containing the carboxyltransferase domain of *ACCase*. The RT-PCR methodology and primers were the same as reported in Kaundun (2010) [22]. All 64 plants were assembled into sensitive and resistant subgroups and their DNA sequences were examined with the Seqman software (Lasergene, USA) in view of identifying the ACCase mutation(s) responsible for resistance to cycloxydim in BG2.

To formally analyse the influence of the threonine and leucine mutations at ACCase codon 1781 on the efficacy of cycloxydim, only plants that were wild at codon 2027 were considered. For each mutation, heterozygous and homozygous plants were grouped. Survival data from the co-segregation experiment was arranged as a 2×2 contingency table for each of the II1781 (homozygous wild type) vs IT1781+TT1781 (heterozygous and homozygous threonine mutants) and II1781 (homozygous wild type) vs IL1781+LL1781 (heterozygous and homozygous leucine mutants) combinations and analysed using Fisher’s Exact test. A p-value of less than 0.05 indicates a statistically significant result at the 5% probability level.

#### Cross resistance to two other DIM herbicides

In order to investigate the cross resistance to two other DIM herbicides commonly used for black-grass control, 48 randomly selected and individually potted BG2 plants each were treated with the recommended field rates of tepraloxydim (100 g ai/ha) and clethodim (120 g ai/ha). For comparison, 16 plants from the standard sensitive population STD1 were treated with the two DIM herbicides. Survival to the herbicides was assessed 21 days after treatment. Prior to herbicide treatment a 1-cm leaf segment was sampled from each plant for DNA analysis at the 1781 and 2027 ACCase codon positions for phenotype to genotype correlation. The resulting data was statistically analysed with the Fisher Exact test in the same way as described for cycloxydim.

#### Level of resistance conferred by mutations at ACCase codon position 1781 and non-target site mechanisms

Sixteen plants with pre-determined wild and mutant genotypes were used to assess the resistance factors associated with non-target site and target site resistance due to the threonine and leucine mutations at ACCase codon position 1781. All plants were wild type at ACCase codon position 2027. The plants consisted of four each of homozygous wild type BG2-II1781, heterozygous mutant BG2-IT1781 and heterozygous mutant BG2-IL1781 from population BG2, and four homozygous wild type STD1-II1781 genotypes from the standard sensitive population STD1. These 16 mother plants were tiller propagated for a period of one year to produce identical tillers for use in the whole plant dose response assays. Tillering was aided by fertilising and trimming the plants regularly. Two genetically identical tillers of each mother plant were sprayed with the following rates of cycloxydim: 1.56, 3.125, 6.125, 12.5, 25, 50, 100, 200, 400, 800 g ai/ha; clodinafop-propargyl: 1.875, 3.75, 7.5, 15, 30, 60, 120, 240, 480 and 960 g ai/ha; pinoxaden: 1.875, 3.75, 7.5, 15, 30, 60, 120, 240, 480 and 960 g ai/ha.

The pots were arranged in a randomized complete block design after herbicide application (blocking per mother plant). Twenty-one days after treatment the tillers were cut above ground level, put into paper envelopes and dried in an oven at 70°C for three days. The dry mass for each individual was recorded to the nearest mg.

Since the mother plants represent the independent biological replicates in the experiment, the dry weight measurements were averaged across technical replicates (tillers) separately for each compound, rate and mother plant, and divided by the average dry weight of the untreated tillers from the corresponding mother plant. GR_50_ estimates were derived from the resulting percent of untreated values separately for each mother plant by a non-linear least-squares regression model described by the equation:
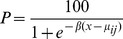
where x denotes log(Rate); µ_ij_ denotes the logGR_50_ for mother plant i of genotype j and β denotes the slope of the rate-response.

The estimated resistance factor (Rf) between a pair of genotypes was calculated as the ratio of the geometric means of the estimated GR_50_ values across mother plants. Confidence intervals around the estimated resistance factors were calculated using the error term derived from an analysis of variance carried out on the logGR_50_ values. All statistical analyses were carried out using SAS software, version 9.2.

#### Establishment of two dCAPS assays for the identification of mutations at ACCase codon position 1781

The dCAPS (derived Cleaved Amplified Polymorphic Sequence) method operates in a 3-step procedure consisting of PCR, restriction digest and gel electrophoresis [23]. It allows selective digestion and differentiation between wild and mutant DNA by the forced introduction of nucleotide change(s) in the dCAPS primer. The dCAPS primers and corresponding restriction enzymes were selected using the freeware developed by Neff et al. (2002) [24]. The first assay consisted of detecting any known mutations at ACCase codon 1781 using the enzyme *Nsi*I whilst the second assay employed the enzyme *Nde*I for the positive identification of a threonine at this position. The recognition sites for *Nsi*I and *Nde*I are ATGCAT and CATATG respectively. The reverse dCAPS primers used in the *Nsi*I and *Nde*I assays were AGAATA**C**GCACTGGCAATAGCAGCACTTCCATGC and CCCTAGAATAGGCACTGGCAATAGCAGCACTTCCATAT respectively. The forced mutations on the primers are underlined. Both assays utilised the same forward primer: ATGACTGACGAAGACCATGATCG. With the first assay, changes in any one of the first two bases of the 1781 codon would disrupt the *Nsi*I restriction site (ATGCAT) and result in an uncut DNA fragment on the agarose gel. In contrast, a restriction site (CATATG) is present for the enzyme *Nde*I when the isoleucine allele is changed into threonine at codon position 1781. Consequently, the presence of a positive threonine allele is manifested as a restricted band on the agarose gel. It is noteworthy that a second forced mutation at position N+31 (in bold) with respect to the first base of the 1781 codon was included on the *Nsi*I primer to eliminate a second non-specific site for this enzyme in black-grass plants. The 48 BG2 and 16 STD1 plants previously sequenced for the entire ACCase CT domain were used to develop the two dCAPS assays. The procedure for the dCAPS assays was as follows: 1 cm leaf segment from each plant was ground on a Spex Certiprep (Metuchen, NJ, USA) 2000 model Genogrinder; DNA was extracted from the ground material on a Beckman Coulter Biomek FX robot (High Wycombe, Buckinghamshire, UK) using a Wizard Magnetic 96 DNA Plant System kit (Promega, Madison, WI, USA). Polymerase chain reactions were carried out using PuReTaq Ready-To-Go PCR beads (Amersham Biosciences, Bucks, UK) in a total volume of 25 µL containing 0.8 µM of each primer and from 10–50 ng genomic DNA and run on an Eppendorf Master Cycle Gradient Thermocycler Model 96 programmed for an initial denaturation step of 94°C of 2 min followed by 40 cycles of 30 s at 94°C, 30 s at 64°C and 1 min at 72°C. A final extension step for 10 min at 72°C was also included. For the positive identification of the wild type isoleucine residue, 8 µl of the PCR product was then digested with 1 µL (5 units) of *Nsi*I at 37°C for 2 hours in a 40 µL reaction. Ten µL of the *Nsi*I treated PCR product was then migrated on a 1×TBE 2% agarose gel containing 0.5 µg mL^−1^ ethidium bromide. The same procedure was employed for the positive identification of the mutant threonine allele, except that *Nsi*I was replaced by the enzyme *Nde*I.

#### Frequency of the T1781 allele in a range of cycloxydim resistant black-grass populations

Forty UK black-grass populations from our cycloxydim resistant collection were screened with 200 g ai/ha cycloxydim in a whole plant assay. Around 50 seedlings were tested per population. Twenty one days after treatment, twelve survivors per population were assessed with the established dCAPS markers described above in order to estimate the relative frequency of T1781 allele among black-grass plants mutated at the corresponding codon. The 480 cycloxydim survivors in all were assayed in a two-step procedure. All plants were screened with the *Nsi*1 based dCAPS marker method. Upon non-digestion of the PCR fragment with *Nsi*1, and thus indication of a mutated allele at codon position 1781, the plants were further tested with the *Nde*I based T1781 specific assay.

## Results

### Resistance Profile of Black-grass Population BG2

All BG2 plants survived the pinoxaden and clodinafop-propargyl treatments while 50±5.8% biomass reduction was achieved with cycloxydim with distinct live and dead plants identified in the pots. As expected, the three ACCase herbicides fully controlled the standard sensitive population. Given that cycloxydim is a poorly metabolisable herbicide, an insensitive target rather than enhanced herbicide detoxification was suspected as the likely mechanism of resistance to this DIM herbicide.

### Selection of Sensitive and Cycloxydim RESISTANT Plants and Identification of Target Site Resistance in BG2

The BG2 population treated with a single rate of cycloxydim at 200 g ai/ha segregated into 21 dead and 27 live plants while all the sensitive STD1 were killed. Sequencing of a PCR fragment encompassing the whole CT domain revealed gene sequences showing over 97% homology with published black-grass ACCase sequences. The sequences were particularly conserved among the 48 BG2 and 16 STD1 plants with only seven nucleotide changes recorded at six ACCase codon positions. Four base changes at codon positions 1801, 1833, 1923 and 1988 were silent. The non-synonymous changes consisted of the commonly reported I1781L and W2027C resistance mutations and a novel I1781T mutation. The I1781L mutation involved a transversion of an adenine to thymine change on the first base of the codon while the I1781T mutation resulted from a transition of a thymine to a cytosine at the second base of the codon. In all, eight different combined 1781/2027 genotypes could be distinguished among the BG2 plants (Table 1). The most common genotypes consisted of wild type sequences for both the 1781 and 2027 codon positions followed by heterozygous mutant IT1781 and IL1781 plants. As expected with highly outcrossing species, compound heterozygotes LT1781 genotypes and plants containing one each of the L1781 or T1781 and C2027 mutant alleles were also identified. It is noteworthy that only a few plants were homozygous for any mutations, thus indicating that the population was very probably at a relatively early stage of resistance selection with ACCase herbicides. Importantly, when the plant genotypes and phenotypes were compared, there was clear evidence that either the T1781 or L1781 alleles were associated with cycloxydim resistance (P<0.001) irrespective of their amino acid status at ACCase codon position 2027. When present alone, a single copy of the mutant cytosine 2027 allele did not confer resistance to cycloxydim.

**Table 1:** Correlation between cycloxydim phenotypes and combined genotypes at ACCase codon positions 1781 and 2027.

Combined 1781 and 2027 ACCase genotypes	cycloxydim 200 g ai/ha number of plants			clethodim 120 g ai/ha number of plants			tepraloxydim 100 g ai/ha number of plants		
	Total	live	Dead	Total	live	Dead	Total	live	Dead
II1781/WW2027	16	0	16	11	0	11	10	0	10
IL1781/WW2027	11	11	0	10	0	10	12	1	11
IT1781/WW2027	9	9	0	6	0	6	5	0	5
TT1781/WW2027	2	2	0	1	0	1	0	0	0
LL1781/WW2027	1	1	0	0	0	0	2	1	1
LT1781/WW2027	2	2	0	1	0	1	3	0	0
II1781/WC2027	5	0	5	12	0	12	7	0	7
IT1781/WC2027	2	2	0	2	0	2	3	0	3
IL1781/WC2027	0	0	0	5	0	5	4	0	4
II1781/CC2027	0	0	0	0	0	0	2	0	2

### Cross Resistance with Two other DIM Herbicides

ACCase gene analysis of the two additional sets of 48 BG2 plants, each treated with tepraloxydim and clethodim revealed genotypic frequencies comparable to the one observed with the cycloxydim treated plants (Table 1) but with two additional combined IL1781/WC2027 and II1781/CC2027 genotypes identified. All 48 plants sprayed with clethodim were controlled regardless of their genotypes at codon positions 1781 and 2027. Similarly, all BG2 individuals with either the T1781 or C2027 alleles were killed with tepraloxydim either alone or in combination with another mutation. One out of 12 IL1781-WW2027 and one out of two LL1781-WW2027 plants survived the tepraloxydim treatment. Formal statistical analysis with Fisher Exact test did not identify a significant difference between wild type and mutant L1781 plants for tepraloxydim (P = 0.493).

### Level of Resistance Conferred to a FOP a DIM and DEN Herbicides

**Figure 1:**
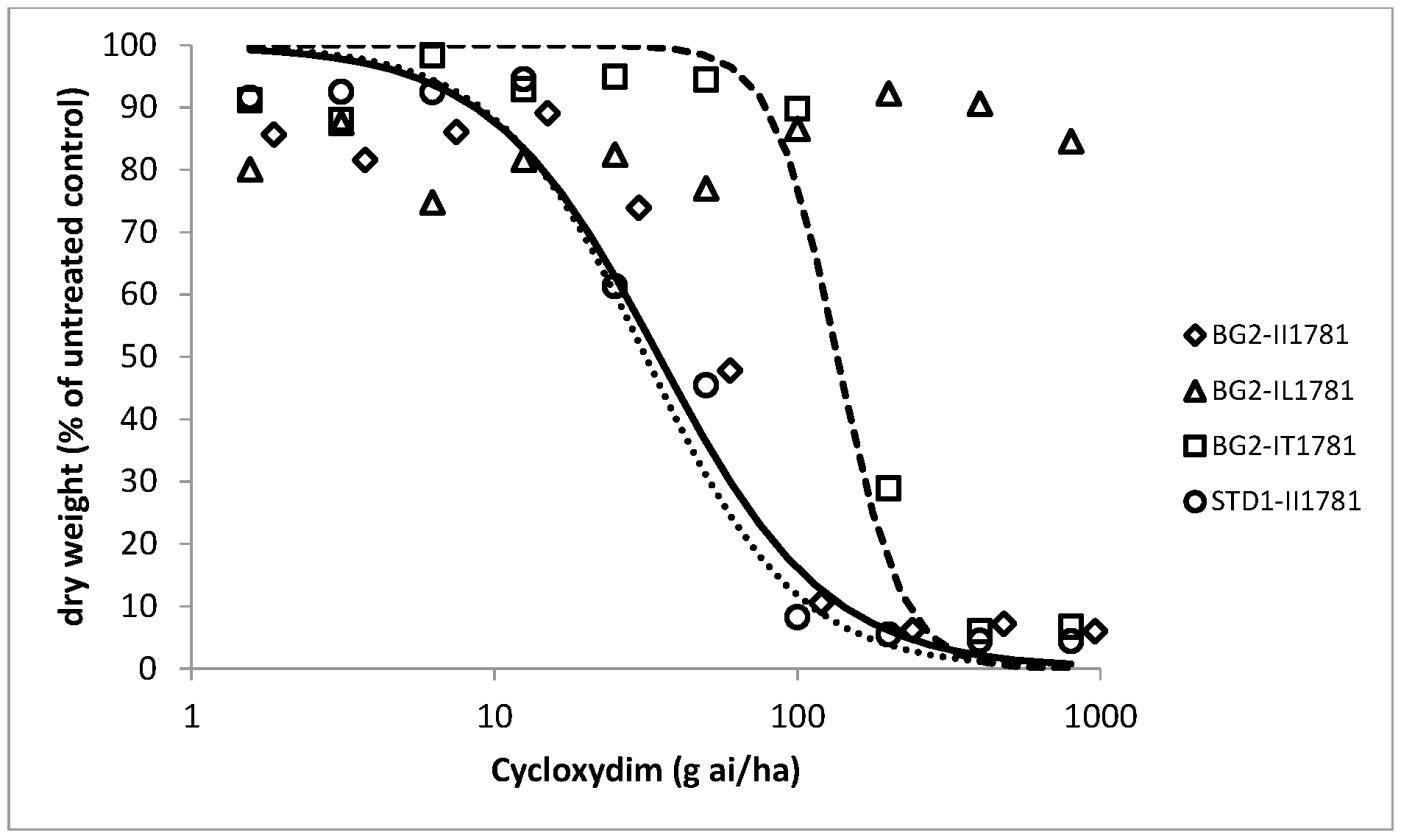
Cycloxydim whole plant dose response tests on four plant groups. Homozygous wild type STD1-II1781 from the standard sensitive population; homozygous wild type BG2-II1781; heterozygous mutant type BG2-IT1781 and heterozygous mutant type BG2-IL1781 from population BG2.

**Figure 2:**
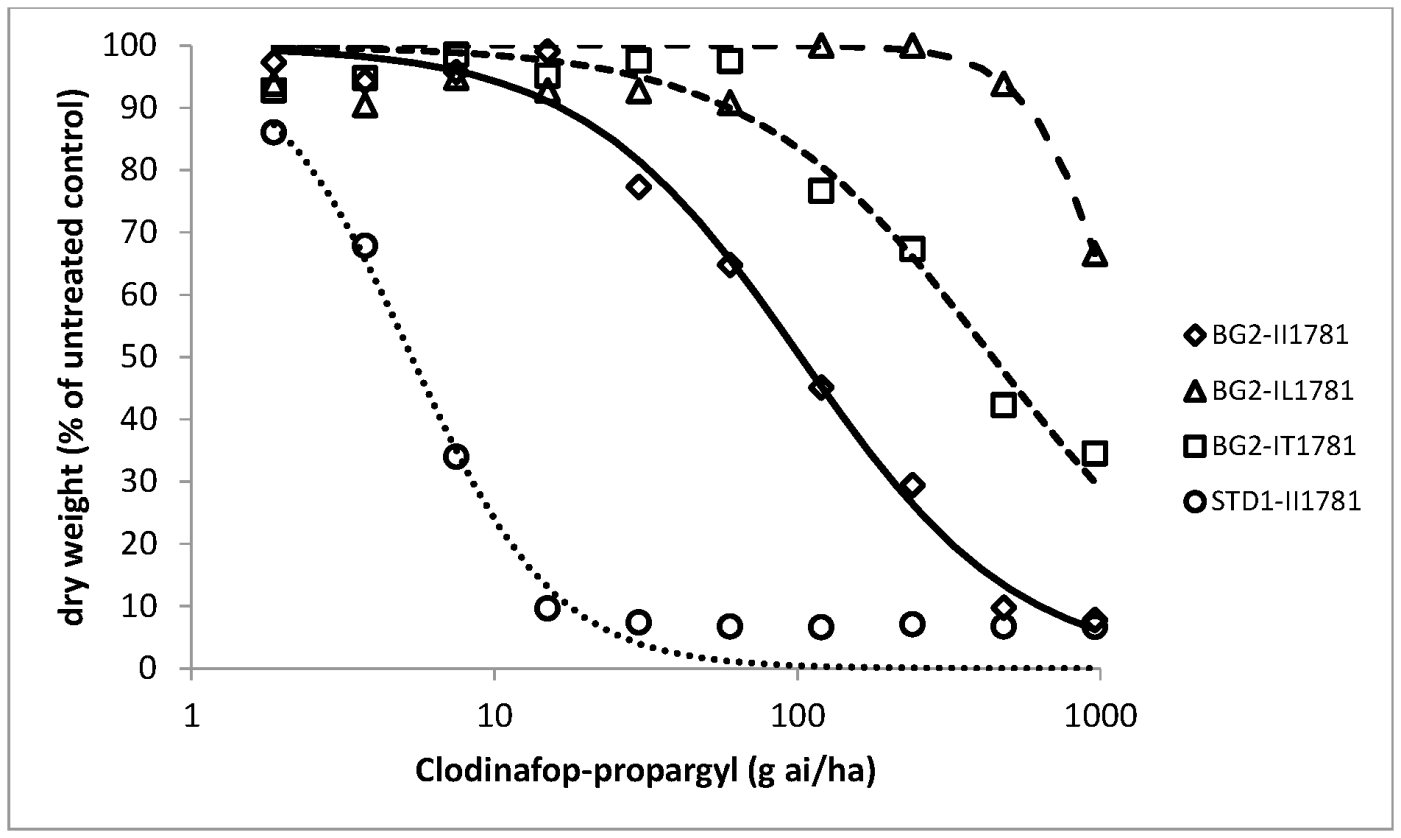
Clodinafop-propargyl whole plant dose response tests on four plant groups. Homozygous wild type STD1-II1781 from the standard sensitive population; homozygous wild type BG2-II1781; heterozygous mutant type BG2-IT1781 and heterozygous mutant type BG2-IL1781 from population BG2.

**Figure 3:**
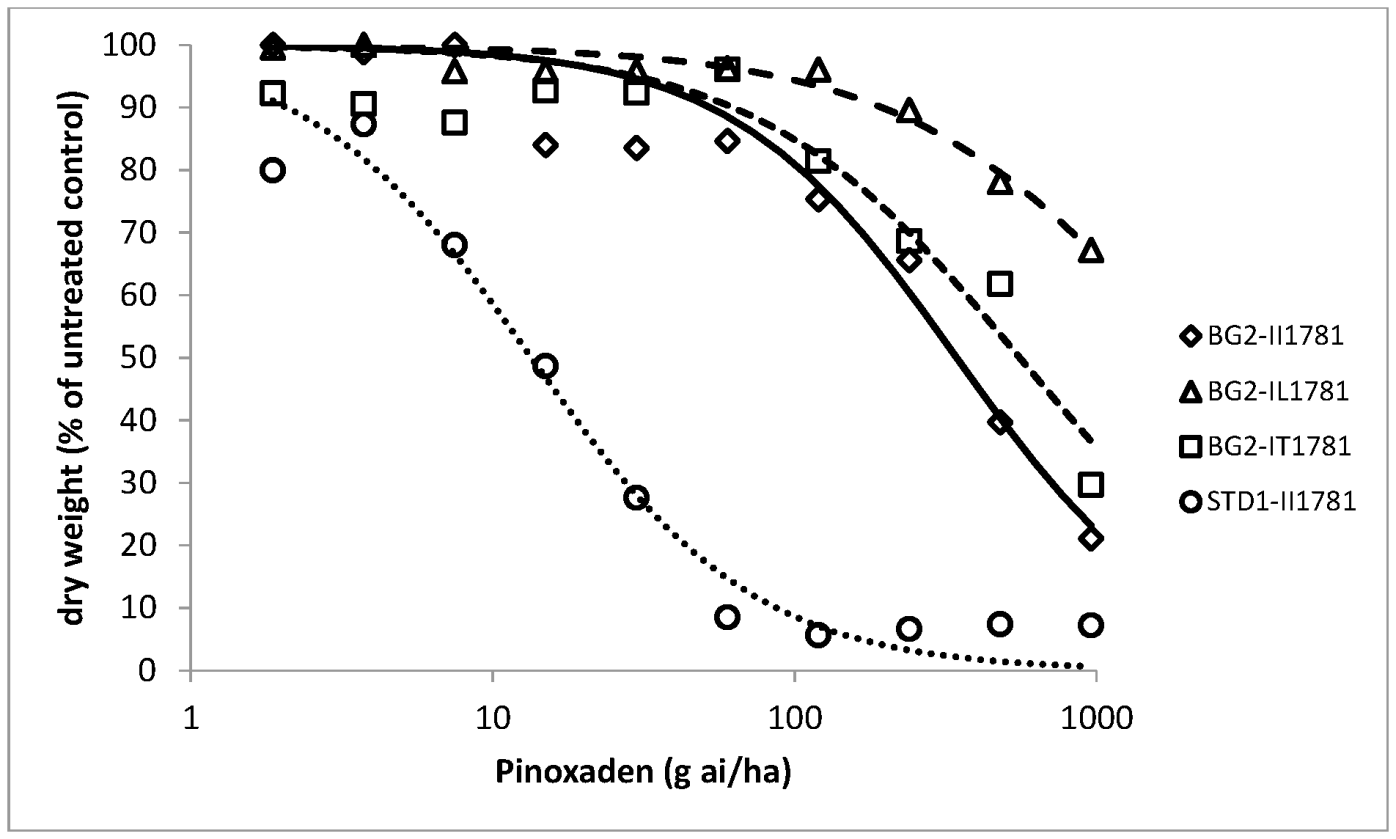
Pinoxaden whole plant dose response tests on four plant groups. Homozygous wild type STD1-II1781 from the standard sensitive population; homozygous wild type BG2-II1781; heterozygous mutant type BG2-IT1781 and heterozygous mutant type BG2-IL1781 from population BG2.

As all BG2 plants were found to be resistant to pinoxaden and clodinafop-propargyl in the initial resistance confirmation tests, dose response assays were conducted on pre-determined wild and mutant genotypes at ACCase codon position 1781 in order to assess the relative importance of target site versus non-target site resistance to these two herbicides as well as cycloxydim. Dose responses could not be obtained or fully exploited for the IL1781 genotypes as the leucine allele conferred very high levels of resistance to all three herbicides (Table 2). This was particularly the case for cycloxydim with a low average biomass reduction of 15% for leucine mutant plants compared to untreated controls at the highest rate tested (Figure 1). Similarly, clodinafop-propargyl and pinoxaden provided a maximum of 35% biomass reduction at the highest rate of 960 g ai/ha (Figure 2 and 3). Comparison of wild II1781 and IT1781 genotypes from population BG2 yielded estimated resistance factors of 4.7 and 4.0 for clodinafop-propargyl and cycloxydim respectively but only 1.6 for pinoxaden (Table 3). As the confidence intervals around this latter estimate contained the value 1, it can be concluded that a single copy of the T1781 allele does not confer resistance to the DEN herbicide. There was no evidence of a shift in the dose response between the wild type BG2-II1781 and STD1-II1781 genotypes for cycloxydim confirming the former co-segregation study which showed that the DIM herbicide is not affected by non-target site resistance in BG2. Significantly, very high levels of non-target site resistance were identified for pinoxaden (estimated Rf = 28) and clodinafop-propargyl (estimated Rf = 18) as determined by comparing the wild type BG2-II1781 and STD1-II1781 genotypes for these two herbicides (Table 3). It is noteworthy that the effects of target site and non-target site mechanisms were additive in negatively affecting these two cereal selective ACCase herbicides. This is illustrated by a resistance factor of 45 for pinoxaden (comparison of wild type STD1-II1781 and mutant BG2-IT1781 genotypes), which is equivalent to the product of the individual target site (Rf = 1.6) and non-target site resistance (Rf = 27.9) contributions.

**Table 2:** Estimated GR50 values for three ACCase herbicides and different genotypes at ACCase codon position 1781.

	STD1-II1781	BG2-II1781	BG2-IT1781	BG2-IL1781
Cycloxydim	39.3 (24.6–62.8)	40.5 (25.3–64.8)	163 (102–261)	>800
Clodinafop-propargyl	5.15 (1.72–15.4)	91.5 (30.6–273.4)	433 (145–1292)	>959
Pinoxaden	11.0 (4.4–27.5)	307 (123–767)	500 (200–1248)	>1091

95% confidence limits in brackets.

**Table 3:** Estimated resistance factors for three ACCase herbicides and four different 1781 ACCase plant groups.

Genotypes	Cycloxydim	Clodinafop-propargyl	Pinoxaden
BG2-II1781 v STD1-II1781	1.0 (0.5–2.0)	17.8 (3.8–83.5)	27.9 (5.9–131)
BG2-IT1781 v STD1-II1781	4.2 (2.1–8.1)	84.0 (17.9–395)	45 (9.6–213)
BG2-IL1781 v STD1-II1781	>20.4	>186	>99
BG2-IT1781 v BG2-II1781	4.0 (2.1–7.8)	4.7 (1.0–22.2)	1.6 (0.4–7.7)
BG2-IL1781 v BG2-II1781	>19.8	>10	>3.6
BG2-IL1781 v BG2-IT1781	>4.9	>2.2	>2.2

BG2- II1781, BG2-IT1781 and BG2- IT1781 originates from the same BG2 population and thus shares the same genetic background except for their amino acids at ACCase position 1781.

STD1-II1781 originates from standard sensitive population STD1 used for comparison.

### dCAPS Assays for Identifying the I1781 and Specifically the T1781 Allele

**Figure 4:**
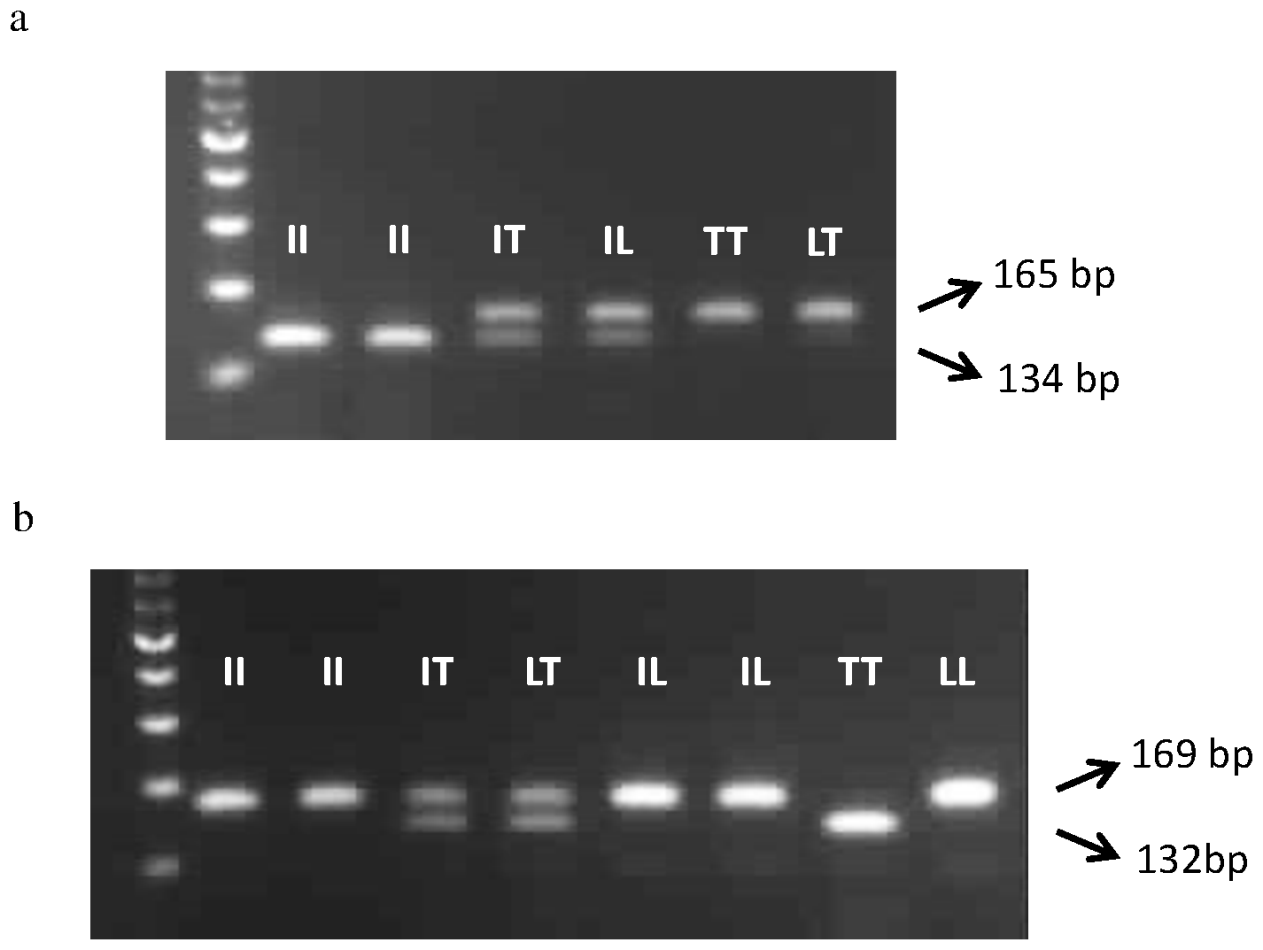
dCAPS procedures for the detection of the wild type isoleucine and mutant threonine amino acid residues at ACCase codon position 1781: (4a) *Nsi*I restricted (134 bp) correspond to the I1781 allele and unrestricted fragments (165 bp) correspond to either T1781 or L1781 alleles. (4b) *Nde*I restricted fragments (132 bp) correspond to the mutant threonine allele while the undigested band (169 bp) corresponds to either the wild type isoleucine or leucine alleles. Heterozygous plants show one each of the restricted and unrestricted PCR fragments in the two assays.

Two dCAPS assays were developed to address the discovery of a novel mutation at ACCase codon position 1781. One of the assays using the enzyme *Nsi*I consisted of detecting any mutation at codon position 1781 and a second *Nde*I assay was employed to specifically identify a threonine residue at this position. Typical dCAPS profiles are provided on figures 4a and 4b. PCR generated fragments of 165 bp and 169 bp for the *Nsi*I and *Nde*I based assays respectively. Upon restriction with the *Nsi*I enzyme, a shorter band of 134 bp was generated for plants containing a wild type isoleucine allele or the AT dinucleotide at the first and second bases of the 1781 codon. Non-digested and undistinguishable fragments were present for either the L1781 or T1781 alleles characterised by the TT and AC dinucleotides respectively. Likewise, with the *Nde*I assay a restricted and shorter band of 132 bp was present for plants that specifically contained the T1781 allele, whilst either the I1781 wild type or L1781 mutant alleles were manifested as non-digested PCR fragments. When used in combination, the two *Nsi*I and *Nde*I assays allowed unambiguous discrimination of all types of wild and mutant 1781 alleles in BG2. Overall, the results from the dCAPS assay was totally corroborated with sequencing data available for 48 BG2 plants and 16 STD1 used in the cycloxydim co-segregation study, thus testifying the robustness of the two dCAPS assays developed here.

### Frequency of the T1781 Mutation in a Wide Range of Cycloxydim Resistant Black-grass Populations from the UK

All the 40 black-grass populations screened showed some levels of cycloxydim resistance ranging from 0% to 75% biomass reduction relative to the untreated control. Testing of 12 survivors per population with the *Nsi*I assay identified at least three 1781 mutant plants per population. Overall the genotypic frequencies were 0.06, 0.66 and 0.28 for homozygous wild type, heterozygous mutant and homozygous mutant types at ACCase position 1781 among the cycloxydim resistant plants. Analysis of the mutant plants with the threonine specific assays showed that only two plants contained the T1781 allele at the heterozygous state. This amounted to a very low T1781 allelic frequency of 0.005 among the 1781 ACCase mutant plants. That an L1781 allele was predominantly present in the samples that were not digested with the *Nsi*I assay was further confirmed via sequencing analysis of a few populations.

## Discussion

Black-grass is a growing threat to arable crop production in the UK. Field cultivation, delayed sowing, higher seeding rate and competitive crop varieties are reactively being practiced due to the evolution of resistance to the most effective single site post-emergence herbicides [25]. Once resistance has evolved, however, long term field experiments have shown that, non-chemical weed control measures, though important for managing the seed bank, will contribute very little to the overall percentage of resistant individuals in the population [26]. Therefore, it is imperative to better understand the basis of herbicide resistance evolution in order to make the best use of existing products and other cultural methods for sustainable black-grass control.

### Target Site Resistance Mutations in BG2

Sequencing experiments showed that the ACCase carboxyltransferase domain is very much conserved in black-grass. This agrees with previous sequence data from three French populations and in which only four non-synonymous ACCase mutations were identified [16]. Of these, three were found to be associated with ACCase target site resistance. The relative conservation of ACCase in black-grass contrasts sharply with *Lolium* species in which as many as seven non-synonymous mutations were found among 12 plants sequenced from a single population [22] and of these only one resulted in resistance to ACCase inhibiting herbicides. Similar observations were made in other ryegrass populations from the UK [27] and Australia [28]. Thus, black-grass appears to be a very suitable species for discovering new ACCase target site resistance mutations in grass weeds. In this respect, four out of the seven ACCase codon positions reported to be involved in an insensitive target were initially identified in black-grass before being found to endow resistance in other grass weed species [29], [30], [31].

Three target site mutations in all were identified in population BG2. These included the W2027C mutation which was detected in *Alopecurus myosuroides* [16] *Avena sterilis* [32], *Lolium* [28], *Phalaris minor* [33] and *Sorghum bicolor* [34]. In black-grass the W2027C mutation was found to confer resistance to the FOP herbicides clodinafop-propargyl, haloxyfop-P-methyl and fenoxaprop-P-ethyl but not to the DIM herbicides cycloxydim and clethodim [35]. Here we observed that the W2027C mutation did not confer resistance to tepraloxydim either. The influence of the W2027C mutation on clodinafop-propargyl and pinoxaden could not be investigated due to the small number of plants that contained this mutation and also potential confounding non-target site resistance present in BG2. Crossing of several heterozygous BG2-WC2027 plants to produce homozygous wild type WW2027 and CC2027 genotypes and subsequent whole plant dose response assays will allow precise estimation of the level of resistance conferred by the W2027C mutation on these two cereal selective ACCase herbicides.

Twenty five percent of BG2 individuals contained at least one copy of the mutant L1781 allele. The role of the I1781L mutation in conferring resistance to a range of ACCase herbicides is amply demonstrated via yeast gene replacement [36], enzyme [37] and molecular [38] assays since the first identification in a *Setaria viridis* population [39], and subsequently in four other major grass weeds including *Lolium* [36], *Avena* [40], *Alopecurus* [41] and *Echinochloa* [42] species. Given the conservation of the ACCase gene among grass species and the absence of a fitness penalty [43], it would not be surprising to find that the I1781L mutation is also involved in the additional 30 or so weed species that have evolved resistance to ACCase herbicides [11].

This study has also identified a novel I1781T mutation found to confer comparatively lower levels of resistance to all three FOP, DIM and DEN herbicides tested. The I1781T present in one copy was found to confer dominant resistance to cycloxydim and clodinafop-propargyl but only low and non-significant levels of resistance to pinoxaden. It is noteworthy that the dose responses were conducted on heterozygous IT1781 plants and that when present at the homozygous state, the mutant threonine allele may confer significant levels of resistance to pinoxaden.

Overall three allelic variants have been identified at position 1781 to date, including the I1781V mutation reported to confer at best relatively low levels of resistance to some ACCase herbicides in a *Phalaris paradoxa* population [44]. However, the precise impact of the valine mutant allele on ACCase herbicides remains to be ascertained as non-target site resistance was overlooked in this population. The lower resistance factors associated with the threonine and valine as compared to the leucine mutant alleles is paradoxical given that leucine amounts to a smaller amino acid change relative to the valine and threonine substitutions. While recent crystallography studies have provided a better understanding of the precise binding of FOP, DIM and DEN herbicides to its target enzyme [45], [46], [47], more remains to be done to better appreciate how subtle amino acid changes can have a profound influence on the efficacies of ACCase herbicides.

### Detection of Mutations at Codon Position 1781

Several DNA based methods have been developed for the quick identification of the I1781L target site ACCase mutation in grass weeds. These include the PASA [38], dCAPS [48], [49], qPCR [50], Taqman [13], Maldi-Tof [51] and SNaPshot [15] assays. These methodologies altogether have contributed towards identifying other mechanisms of resistance to ACCase herbicides by quickly disregarding populations characterised by the I1781L mutation. Additionally, they have permitted the investigation of gene flow [52] and mode of evolution of ACCase target site resistance [53]. However, they are all ambiguous in the light of the present discovery of the I1781T mutation in that they have been designed to detect a nucleotide change on the first base of the 1781 nucleotide triplet only. Thus, in so doing they would have missed other mutations resulting from a nucleotide change on the second base of the 1781 triplet. The assay developed here is very similar to the one published previously [48] except that the dCAPS primer ends on the third base of the 1781 codon to take into account the possible nucleotide changes that can occur on the first and also second base of the codon triplet. It is to be noted, however, that further confirmation and differentiation between all the possible allelic variants would require other assays such as the one developed here for positively identifying the threonine mutant at codon position 1781.

### Rarity of the I1781T Mutation

The I1781L mutation has been widely identified in several hundred grass weed populations worldwide [13], [15], [29]. In contrast, the I1781T has only recently been discovered in population BG2. The rarity of the threonine 1781 allele could be due to the lower level of resistance it confers to ACCase herbicides as compared to the L1781 mutation, making it less probable to be selected under ACCase herbicide selection pressure. However, the T1781 allele did confer dominant resistance to clodinafop-propargyl and cycloxydim, two widely used herbicides in the UK. Therefore both L1781 and T1781 alleles should have been selected to equal degrees in black-grass populations. The seldom encounter of the T1781 allele could be explained by the fact that all high throughput DNA based assays employed to date were ambiguous and may have missed the second nucleotide change that results in the substitution of an isoleucine to the threonine allele. The analysis of a large number of cycloxydim resistant black-grass samples using an unambiguous assay developed here has revealed that the T1781 allele is indeed a very rare event accounting for only 0.5% of mutations that occur at position 1781. Alternatively, the rarity of the I1781T could be due to a fitness cost associated with the threonine allele as compared to the leucine 1781 allele [43]. That dissimilar amino acids can confer different levels of fitness at the same codon position is illustrated by changes at the 2088 ACCase codon. The C2088R mutation appeared to be affected by a fitness cost in *Lolium* species based on initial enzyme data [28] whilst the C2088F is fixed in several species such as *Bromus* and even *Hordeum* and by definition is not affected by a fitness penalty [54].

### Non-target Site Resistance Mechanisms in BG2

Population BG2 was completely resistant to clodinafop-propargyl and pinoxaden. Yet 25% of the plants did not have a mutation in the herbicide binding carboxyltransferable domain, allowing indirect inference of the involvement of non-target site based mechanisms in BG2. Non-target site resistance has previously been found to affect the metabolisable FOP and DEN herbicides in black-grass [55]. In a survey of over 200 French black-grass populations, an overwhelming 75% of resistant plants were found not to contain a known ACCase mutation [51]. The widespread occurrence of non-target site versus target site resistance to ACCase herbicides was upheld when the whole range of the species was investigated [13]. The frequency of non-target site resistance established by genotyping plants with SNP markers at known resistance codon positions should be taken with caution. DNA markers used to screen resistant populations are sometimes ambiguous, as demonstrated here, and novel mutations yet to be identified may be present in populations classified as non-target site resistant. Also, it has long been assumed that non-target site resistance and in particular, metabolic resistance, confers lower levels of resistance compared target site resistance. Here, we showed that the levels of resistance to clodinafop-propargyl and pinoxaden due to non-target site mechanisms were higher than the I1781T and, potentially, the I1781L mutations. Precise assessment of the level of non-target site resistance was possible by comparing wild type plants from BG2 and the standard sensitive population. If the target site mutant IT1781 subpopulation was directly compared to the standard sensitive population, as with many previous studies, then it would have been wrongly concluded the I1781T mutation conferred 45-fold resistance to pinoxaden. As established, this target site mutation contributed very little to pinoxaden resistance and that failure to control BG2 is largely attributed to non-target site mechanism(s).

### Differential Efficacies of ACCase Herbicides and Management of Black Grass in Rotational Broadleaved Crops

Resistance to ACCase herbicides has been classified as FOP or DIM specific [16] though differences exist among compounds from the same chemical sub-class [15]. In a survey of black-grass populations from France for instance, increasing levels of control were observed from fenoxaprop-P-ethyl to pinoxaden through clodinafop-propargyl [55]. Similarly in this study, the non-metabolisable DIM herbicides showed different levels of efficacies on the black-grass population BG2. Cycloxydim killed all plants characterised by non-target site resistance and the W2027C ACCase mutation. Tepraloxydim was very effective at controlling all plants characterised by non-target site resistance, the I1781T and W2027C mutations and most individuals with the commonly encountered I1781L mutation. The competitive edge of tepraloxydim over cycloxydim may explain why the former is gradually supplanting the latter for controlling ACCase resistant black-grass populations in the UK. However, a few plants containing the I1781L mutation were resistant to tepraloxydim in such a way that over time increased use of this herbicide will select for homozygous LL1781 resistant plants and result in product failure. Clethodim was by far the best herbicide, providing 100% kill on all wild and mutant ACCase genotypes. Black-grass plants containing the D2078G ACCase mutation [16] and C2088R mutation (yet to be identified in this species), are known to affect clethodim [28], [56]. Consequently its impending use for black-grass control in dicotyledonous rotational crops in the UK could lead to these mutations being favourably selected. It is imperative, therefore, that grass weed control should not only be carried out in conjunction with diverse herbicide modes of action but should also proactively include non-chemical weed control methods for prolonging the sustainability of DIM herbicides that are still very effective at controlling a significant proportion of black-grass populations.

### Conclusion

This study highlights the importance of segregating grass weed populations into individual components for establishing the role of target site and non-target site mechanisms in conferring resistance to ACCase inhibiting herbicides. In addition to two known target site resistance mutations, a novel I1781T mutant ACCase allelic variant was identified and its importance on ACCase herbicide efficacies was precisely assessed in a black-grass population. The study also showed that contrary to common belief, accumulation of non-target site resistance in single plants and populations over the years has, in some cases, generated levels of resistance that are higher than the most common target site mechanisms.
